# Supplementary material for: Harnessing digital health to objectively assess cancer-related fatigue: The impact of fatigue on mobility performance
Source: PLoS One. 2021 Feb 26;16(2):e0246101. doi: 10.1371/journal.pone.0246101 (PMC7910036; doi:10.1371/journal.pone.0246101)
Supplement: Attachment — Submitted filename: Response letter PlosOne CRF-Final_rev.docx [file pone.0246101.s002.docx]

| Original Title | Harnessing Digital Health to Objectively Assess Cancer-Related Fatigue: The Impact of Fatigue on Mobility Performance |
| --- | --- |
| Manuscript ID | PONE-D-20-01496 |

**Authors’ response to the reviewer**

Dear Editor,

We appreciate the constructive comments and suggestions made by the academic editor and reviewers. Overall, where relevant, we have made changes that we believe are responsive to comments, and which enhance the quality of the manuscript. Specific modifications to the manuscript in response to comments by the academic editor are highlighted using Word Text Highlight Color in the manuscript, and the updated locations in the manuscript are specified.

In the revised draft we:

- Addressed the reviewers’ concerns and critiques
- Performed data analysis based on reviewer’s critique
- Edited the Discussion section to better interpret the key findings
- Modified figures and tables
- Added the current challenges in remotely assessing cancer patients during COVID-19 to the introduction

None of the co-authors, claimed any relevant conflict of interest. Partial support was provided by the National Institutes of Health/National Cancer Institute (award number 1R21CA190933-01A1) and the National Institutes of Health/National Institute on Aging (award number 1R42AG060853-01). The content is solely the responsibility of the authors and does not necessarily represent the official views of sponsors. The data used in this study would be available upon request. In addition, the data was uploaded on PhysioNet. Submission ID: WusVI9kAAbbcKZrED4MK, which is currently under review.

Thank you for your attention and consideration. We look forward to your final review.

Sincerely,

Yvonne H. Sada, Olia Poursina, He Zhou, Biruh T. Workeneh, Sandhya V. Maddali, Bijan Najafi

| **Journal Requirements** | **Revision and reply to the queries** |
| --- | --- |
| **C1.** Please ensure that your manuscript meets PLOS ONE's style requirements, including those for file naming. The PLOS ONE style templates can be found at <https://journals.plos.org/plosone/s/file?id=wjVg/PLOSOne_formatting_sample_main_body.pdf>  andhttps://journals.plos.org/plosone/s/file?id=ba62/PLOSOne_formatting_sample_title_authors_affiliations.pdf | We have reformatted the manuscript to meet the style requirements. |
| **C2.** Thank you for including your ethics statement: "Informed consent approved by local Institutional Review Boards was obtained from all participants prior to screening." Please amend your current ethics statement to include the full name of the ethics committee/institutional review board(s) that approved your specific study. Once you have amended this/these statement(s) in the Methods section of the manuscript, please add the same text to the “Ethics Statement” field of the submission form (via “Edit Submission”).  For additional information about PLOS ONE ethical requirements for human subjects research, please refer to <http://journals.plos.org/plosone/s/submission-guidelines#loc-human-subjects-research>. | We have revised the Methods section to clarify the Institutional Review Board (**see page7, lines 132-133)**  *Informed consent was obtained from all participants prior to screening. This study was approved by the Baylor College of Medicine Institutional Review Board*. |
| **C3**. Thank you for stating in your Funding Statement:  "Partial support was provided by the National Institutes of Health/National Cancer Institute (award number 1R21CA190933-01A1). "  Please provide an amended statement that declares *all* the funding or sources of support (whether external or internal to your organization) received during this study, as detailed online in our guide for authors at http://journals.plos.org/plosone/s/submit-now. Please also include the statement “There was no additional external funding received for this study.” in your updated Funding Statement. Please include your amended Funding Statement within your cover letter. We will change the online submission form on your behalf. | We have revised the Funding Statement in our cover letter as well as the manuscript.(page 16 lines 331-334)  *“This study was supported by the National Institutes of Health/National Cancer Institute (award number 1R21CA190933-01A1), the National Institutes of Health/National Institute on Aging (award number 1R42AG060853-01), and internal support from Baylor College of Medicine. There was no additional external funding received for this study. The content is solely the responsibility of the authors and does not necessarily represent the official views of sponsors."* |
| **C4.** In your Data Availability statement, you have not specified where the minimal data set underlying the results described in your manuscript can be found. PLOS defines a study's minimal data set as the underlying data used to reach the conclusions drawn in the manuscript and any additional data required to replicate the reported study findings in their entirety. All PLOS journals require that the minimal data set be made fully available. For more information about our data policy, please see <http://journals.plos.org/plosone/s/data-availability>.  Upon re-submitting your revised manuscript, please upload your study’s minimal underlying data set as either Supporting Information files or to a stable, public repository and include the relevant URLs, DOIs, or accession numbers within your revised cover letter. For a list of acceptable repositories, please see http://journals.plos.org/plosone/s/data-availability#loc-recommended-repositories. Any potentially identifying patient information must be fully anonymized.  Important: If there are ethical or legal restrictions to sharing your data publicly, please explain these restrictions in detail. Please see our guidelines for more information on what we consider unacceptable restrictions to publicly sharing data: http://journals.plos.org/plosone/s/data-availability#loc-unacceptabledata-access-restrictions. Note that it is not acceptable for the authors to be the sole named individuals responsible for ensuring data access.  We will update your Data Availability statement to reflect the information you provide in your cover letter. | The minimum data to replicate the results of this study was also uploaded as a Supporting Information file. Additional data not directly related to the results of this study (e.g., additional clinical and demographic information) would be available upon request. The cover letter was updated accordingly. |

| **Reviewer #1** | **Revision and reply to reviewer’s comment** |
| --- | --- |
| **R1.C1.**  Abstract: The first sentence of the objective implies that CRF is a comorbidity of CIPN, which I don't think is the intention. Please modify this first sentence and section to reflect the primary aims of the study to evaluate mobility in CRF (regardless of comorbidity) and clarify in the methods that this is a secondary analysis of a clinical trial of exercise in CIPN. | Thank you for this comment. We agree the abstract was unclear. We have edited the abstract Objective and Methods sections to reflect these changes. |
| **R1.C2.**  Additionally, a minor point, but please report 'sex' (physical/biological construct) rather than 'gender' (social construct) unless you are referring to the identity of individuals. | We have replaced the term “gender” by ‘sex’ as advised. |
| **R1.C3.**  Introduction: 2nd paragraph, 1st sentence - please expand on the impact of CRF on treatment decisions (i.e. dose delays/modifications during treatment? treatment decisions only during survivorship?) to illustrate the complete impact of CRF. | We agree with this comment and have edited the Introduction to reflect the broad impact CRF may have on patients throughout the cancer continuum from cancer treatment to survivorship (**page 4, lines 69-71)**.  *CRF is an important component of cancer-related treatment decision making, such as treatment intensity, dose modifications, referral for psychosocial support, or exercise interventions [**7**,* *8**]*. |
| **R1.C4.**  Introduction: 3rd paragraph, last sentence - please change to 'Due to the limitations of current CRF assessments tools, improved assessment methods are needed'. No data exist to suggest that objective/quantifiable measures will be inherently better/more valid/more sensitive/more reliable in assessing CRF. | Thank you for this comment. We have revised the last sentence of the third paragraph in the Introduction (**page 5, lines 82-83**). |
| **R1.C5.** Introduction: 4th and 5th paragraphs - I'd reorganize these paragraphs for clarity in the following manner (essentially flip paragraphs 4 and 5): 1) links between physical activity and CRF and rationale for evaluating CRF; 2) prospective utility of digital biomarkers in evaluating mobility. At present, the utility of biomarkers in evaluating physical activity is presented before a clear rationale for evaluating physical activity in relation to CRF has been established. | Thank you for this great suggestion. We have changed the order of paragraphs 4 and 5 (**pages 5-6, lines 84-109).** |
| **R1.C6.**  Introduction: 5th paragraph, final sentence - This is a cross-sectional study, and your hypothesis should reflect this. '...CRF may >>BE ASSOCIATED WITH<< increased sedentary behavior, decreased locomotion...' Please refrain from implying causation. | We have changed the last sentence of the final paragraph of the Introduction to reflect this comment (**page 6, line 114-115)**.  *“Thus, the primary objective of this study is to examine the association between CRF and mobility performance, as well as motor capacity, measured by wearable sensors. Our second hypothesis is that CRF may be associated with increased sedentary behavior, decreased locomotion, and reduce the duration of standing posture”* |
| **R1.C7.**  Methods: As a general point, the wide range of available assessments is a strength of this cross-sectional assessment, as it allows for analysis of the wide range of confounders. However, assessments that are not relevant to the primary aims can be describe more concisely (i.e. 'Plantar numbness severity was evaluated by VPT as per prior studies [26, 27] and using established thresholds [25, 26]). | Thank you for this comment. We have revised the Methods “Demographic and Clinical Characteristics” section to describe assessments for plantar numbness severity, depression, cognitive impairment, and concern for falling more concisely (**page 7, line 141-143**). |
| **R1.C8.**  Statistical Analysis: The ANCOVA should also account for the significant between-group differences in FES-I scores, as an increased fear of falling is associated with decreases in mobility | Thank you for this comment. For DPA data, we adjusted results for age, BMI, and FES-I. The Methods “Statistical Analysis” section (**page 10, line199**), and Table 3 have been revised to reflect the new analysis. |
| **R1.C9.**  Results: This section reveals the critical flaw in this study (albeit one that is easily addressable) in paragraph 3 - demographics/clinical characteristics are presented and inter-group differences are calculated for 36 subjects, while DPA data are only available for 28 subjects. Given that the aims and conclusions are all based on DPA data, demographic data and inter-group differences MUST be presented and calculated based on the group of subjects with available DPA data to allow for sufficient analysis of potential confounding factors. This manuscript currently presents demographic/clinical characteristic data (to determine confounders) and DPA data as though they are from the same cohort when they ARE NOT! The authors must address this critical flaw to facilitate valid analysis of data. | Thank you for this comment. We have performed all analyses using only the 28 patients with complete DPA data and have revised the Methods “Participants” section, (**page 6, line117**), first and second paragraph of the Results (**pages 10-11, lines 215-229** ), as well as Tables 1 and 2 to reflect the new cohort. |
| **R1.C10.**  Results: Paragraph 1, sentence 5 - a cross sectional analysis of 36/28 people cannot be used to suggest that CRF is independent of age, ethnicity and race. Please modify along the lines of ' age, ethnicity and race did not significantly differ between groups.' | Thank you for this comment. We have changed the sentence in the Results paragraph 1 (**page 11, lines 222-223)**.  *Age, ethnicity, and race did not differ significantly between the two groups.* |
| **R1.C11.**  Results: Paragraph 1, sentence 7 & 8 – please refrain from reporting non-significant results. The data show that no inter-group differences in self-reported pain levels, plantar numbness, depression, and prescription medications exist. | We removed the non-significant results in the first paragraph of the Results (**page 11, line 222)**. |
| **R1.C12.**  Results: Paragraph 2, sentence 3 - again, these results are all non-significant. There are no differences in gait speed and static balance between groups. Please delete or revise this sentence. | Our initial hypothesis was “CRF will impact mobility performance without a noticeable impact on motor capacity”. Thus, we kept the following sentence in the Results section, paragraph 2:  *In addition, aligned with the initial hypothesis, there were no between group differences regarding motor capacity metrics (i.e, habitual walking speed, dual task walking speed, fast walking speed, and static balance).* *(****page 11, lines 227-229)****.* |
| **R1.C12.**  Table 2 - the analysis of between-group differences in frailty in inappropriate. The point of interest here is a difference in the *distribution* of frailty classifications between groups, not individual comparisons of percentages of robust/pre-frail/frail subjects. Accordingly, a chi-square test should be use to test for a difference in this distribution. It appears that these distributions are different (CRF group more frail), which, if validated by appropriate statistics, is a massive confounder for reported results. | We appreciate the comment. We assessed between group differences for distribution of frailty classification and no significant difference was observed (p=0.0108). This was highlighted in the revised text **(page 11, line 226).** |
| **R1.C13.**  Discussion: This section is liable to change quite significantly when appropriate analysis methods are used, so have must made a few comments. In general, a revised discussion should include a greatly expanded impact of the impact of confounders (as calculated through revised statistical analyses). | The results have been updated based on new analyses. Adjustment by FES-I did not noticeably change the results. We acknowledge the limitations of this study in the discussion section including small sample size. We recognize that our sample size is underpowered to address all potential confounders in this study. We have however adjusted results by potential key confounders including age, BMI, and concerns for falls and also reported the effect size which could be used to estimate needed sample size to validate observation of this study in a follow up study. |
| **R1.C14.**  Discussion: Paragraph 2, sentence 1 - again, please refrain from implying causation. CRF is associated with increased sedentary behavior, lower step counts, and shorter walking bouts. | We have revised the Discussion paragraph 2, sentence 1 (**page 12, line 259-260**):  *Our data suggest that CRF is associated with increased sedentary behavior, higher cumulative sedentary postures, lower step counts, and shorter walking bouts.* |
| **R1.C15.**  Discussion: Paragraph 2, sentence 4 - the authors mention the assessment of physical activity levels using accelerometers as a limitation to previous studies, but also a strength of a different prior study in sentence 5. please revise. | We apologize for the lack of clarity. This section of the Discussion was rephrased as the following: (**page 13, lines 265-271**):  “*Previous cross-sectional studies that showed no correlation between fatigue and sedentary behavior are often limited by the use of self-report questionnaires [50]. Few negative studies used objective measurements to quantify physical activities and examined association between sedentary behavior and psychosocial metrics including depression, quality of life, and anxiety in cancer survivors [51-53].* *However, these studies did not use a validated instrument to quantify CRF, and fatigue status was determined using an indirect method such as a sub-component of the quality of life questionnaire.”* |

| **Reviewer #2** | **Revision and reply to reviewer’s comment** |
| --- | --- |
| **R2.C1.**  Materials and Methods: There was no sample size calculation for the study or power of study from the sample size was discussed | We have clarified in the Methods section that this study was a secondary analysis of a parent clinical trial (**page 6, line 118**). Thus, a priori sample size calculation was not done. We added this to the limitations of our study (**Page 15, lines 308-310**).  “…*Because this study is a secondary analysis, the study population was limited to cancer survivors with CIPN, and the study was not powered to clinically validate association between CRF and mobility performance metrics…”* |
| **R2.C2.**  Materials and Methods: The mode of administration for all the questionnaire/inventories to be clearly stated. i.e. self-administered or by assessor/interviewer. | We have added a sentence to the end of the Methods “Demographic and Clinical Characteristics” section to address the reviewer’s comment (**page 7, line 148-149**).  *“All questionnaires were administered by the assessor.”* |
| **R2.C3.**  Statistical analysis: Analysis of Chi-square to be written as chi-square test. If it was referred to the chi-square function presented in the analysis output of a statistical test, the statistical test to be stated. Spearman coefficient to be written as Spearman rank correlation coefficient | The Methods “Statistical Analysis” section has been revised with this correction (**page 10, lines 204& 210-211**). |
| **R2.C4.**  Results: Page 11, for the ‘Results suggest that CRF is independent of age, ethnicity and race (p>0.050), more detail results to be provided in a table form. | Please see Reviewer 1, Comment **R1.C9.** The original statement did not correctly describe the findings in Table 1. Multi-variate logistic regression was not performed due to small sample size. The first paragraph of the Results has been revised (**page 11, lines 222-223**).  *“Age, ethnicity, and race did not differ significantly between the two groups.”* |
| **R2.C5.**  Results: Page 11, for the ethnicity and race, although they have different meaning, can these two be combined and placed as one variable? | Because race is a biologic construct associated with physical characteristics and ethnicity is a cultural construct, we have not combined these two categories into one variable. |
| **R2.C6.**  Results: Page 11, for the higher number of prescription medications (50%, p=0.052), the data to be presented in Table 1.  **R2.C7.**  Results: Page 11, for the section ‘Results suggest that CRF is independent of age, ethnicity and race (p>0.050). The CRF group also showed significantly higher BMI (p=0.017) and lower FACT-G score (p=0.004) than the non-CRF group. Average self-reported pain level (assessed using a subcomponent of FACIT-G) had a trend towards higher levels in the CRF group compared to non-CRF (p=0.060). Participants in the CRF group had a non-significant trend towards higher plantar numbness quantified by VPT on average by 29% (p=0.160)’, the figures to be written other than the p value or the figures to be omitted if the figures were listed in the table (for standardization purposes). | Thank you for this comment. The “50%” was reported incorrectly. The sentence was deleted based on Reviewer 1, Comment **R1.C10** , which recommended deleting non-significant results from the paragraph.  Thank you for this comment. The majority of the quoted Results section (**page 11, line 220-224**) has been changed based on new analysis recommended by Reviewer 1 and previously addressed Comments #R2.C4 and #R2.C6 from Reviewer 2.    “*The average FACIT-F score of the CRF group was significantly lower than the non-CRF group (p<0.001). The CRF group also had a significantly lower FACT-G score than the non-CRF group (p=0.027).* *Age, ethnicity, and race did not differ significantly between the two groups. There were no women in the CRF group, but the non-CRF group was 42% female (p=0.035).”* |
| **R2.C8.**  Results: Page 11, the statement ‘Participants in the CRF group had a non-significant trend towards higher plantar numbness quantified by VPT on average by 29%’ not clear and figure(s) to be presented in Table 1. | We apologize this sentence was not reported correctly. The sentence has been deleted based on Reviewer 1 comment to remove non-significant results from the manuscript text. The figures reported in Table 1 have changed due to new analysis recommended by Reviewer 1. |
| **R2.C9.**  Results: For Table 1, 2, 3, mean, sd and statistical test(s) to be denoted in the table footnote. | The mean and S.D. have been added to the tables. Because statistical analyses were described in the Methods section, we did not repeat this information in the Results section including tables.  Note: Values are mean ± SD |
| **R2.C10.**  Results: For Table 1, 2, n to be stated apart from percentages. The analysis involving categorical variables need to be re-looked. The use of chi square test not clear. Chi-square test is to be employed for categorical data rather than using proportions testing on the variable individual level. | Please note that the unit of some of variables are reported as “%”, which means % of sedentary behavior that is calculated based on % of 24-hours activity monitoring. This does not necessarily mean that these variables are categorical. Based on the recommendation of Reviewer 1, we performed additional statistical analysis and updated the statistical analysis section (**page 10, line 199**). |
| **R2.C11.**  Results: For Figure 2, r symbol was used. Was Pearson's correlation coefficient used in the analysis? If it was meant for Spearman's correlation, the symbol to be replaced with symbol rho. | We apologize for our oversight. Spearman’s rank correlation coefficient was employed in this manuscript. Figure 2 and text were revised to state “rho”. |
| **R2.C12.**  Results: The percentage figures in the text and tables (Table 1, 2, 3) to be at least one decimal point. | The percentage figures have been revised in Tables 1, 2, and 3 to include at least one decimal point. |
| **R2.C13.**  Discussion: Page 17, it was stated 'The sample size was small'. Based on what evidence/ground, this statement was derived? | As stated for Reviewer 2 Comment R1.C1, this study is a secondary analysis and may be underpowered to confirm association between CRF and mobility performance. We revised the limitation section to reflect this **(page 15, lines 308-310).**  “*Because this study is a secondary analysis, the study population was limited to cancer survivors with CIPN, and the study was not powered to clinically validate association between CRF and mobility performance metrics*. “ |
| **R2.C14.**  Discussion: Page 14 and 15, for the statement 'although we adjusted for several factors such as age, pain level, comorbidities, and depression, we were unable to account for confounding factors such as cancer type, chemotherapy regimen, or dosing. In order to address this limitation, we created three separate regression models.' were these for this study or other study? | Thank you for the comment. We deleted the statement from the limitation section in the Discussion. We initially discussed performing regression models but did not present the data because the number of patients was too small, particularly after performing the new analysis per Reviewer 1, which now includes a cohort of 28 participants. Regression models will be planned for future studies in a larger cohort. |
| **R2.C15.**  Discussion: References did not conform with the journal format. | We apologize for oversight. We used Endnote style for PLOS One and updated the format. |

| **Reviewer #3** | **Revision and reply to reviewer’s comment** |
| --- | --- |
| **R3.C1.**  As we know, CRF includes physical fatigue, affective fatigue and cognitive fatigue. Not clear which types of CRF can the wearable pendant sensor measure? | To determine fatigue, we used FACIT questionnaires, which are some of the more commonly used questionnaires in national and international research settings to quantify fatigue in those with chronic health conditions, including cancer patients. The FACIT Fatigue Scale is a short, 13-items, easy to administer tool that measures an individual’s level of fatigue during their usual daily activities over the past week. Our current study is limited to determine association between CRF and mobility performance metrics as a potential digital biomarker of CRF and measurable using wearables. While, we recognize the value of distinguishing different types of fatigue, this is beyond the scope of the current study. |
| **R3.C2.**  Please describe the measuring timepoint of FACIT-G, only at baseline? | This was a cross-sectional study, so the FACIT-G was only measured at baseline. |
| **R3.C3.**  Which is the purpose of this study? the effect of CRF on mobility performance? or the accurancy of the wearable pendant sensor? Please clear the purpose which influence the study design directly. | The last paragraph of the Introduction has been revised to clarify the objectives (**page 6, lines 112-115).** The ultimate goal of this study is to determine potential digital biomarkers which could be served as a surrogate of CRF.  *“We hypothesize that CRF may impact mobility performance without a noticeable impact on motor capacity.* *Thus, the primary objective of this study is to examine the association between CRF and mobility performance, as well as motor capacity, measured by wearable sensors. Our second hypothesis is that CRF may be associated with increased sedentary behavior, decreased locomotion, and reduce the duration of standing posture*” |
| **R3.C4.**  Why “the participants age 55 years or older”? any basis? | This is a secondary analysis of a clinical trial that evaluated the benefit of exercise on adult cancer survivors with chemotherapy-induced peripheral neuropathy (CIPN) who had completed chemotherapy treatment (ClinicalTrials.gov Identifier: NCT02773329) (**page 6, line 120**). Inclusion and exclusion criteria were defined based on primary objectives of the parent study. We have highlighted this as one of the limitations of this study (**page 15, line 307**). |
| **R3.C5.**  “Thirty-six adult cancer survivors with CIPN (age=65.7±9.4 years old, BMI=27.6±4.4kg/m2, gender=36% female), who completed chemotherapy treatment, were recruited.” should be put in the result parts. | Thank you for this comment. This statement has been deleted from the Methods section and added to the first paragraph of Results (**see page 10, line 215**). The number of patients in the cohort has changed based on revised statistical analysis recommendations from Reviewer 1. |
| **R3.C6.**  “several subjects did not wear the sensor for 48-hours”, why? If they won’t wear the pendant over 48 hours, how can we use this device to assess the CRF in the daily care of cancer patients? | Because of battery problems, some subjects did not have a 48-hour recording. In order to have the maximum subjects available, we limited the minimum recording to 24-hours continuous monitoring instead of 48-hours continuous monitoring. Although another study is needed to confirm the reliability of our measurement, fatigue is a dynamic metric and could change daily. Thus, we still believe the proposed study may assist to determine a digital metric surrogate of fatigue that can provide dynamic, objective information rather than patient self-report on fatigue during clinical evaluation. We have an ongoing study in which we are hoping to determine day to day fluctuations in fatigue level. |
| **R3.C7.**  Limitations may expand after the above comments are addressed. | The limitations section was revised to acknowledge the limitations from duration of monitoring **(page 15, lines 310-314).**  ***“****The duration of physical activity monitoring was limited to 24-hours, which may not be sufficient for a reliable assessment of mobility performance. In addition, this study linked the physical activities to self-report fatigue over the week precedent, which may not represent fatigue during the period of physical activity monitoring as fatigue levels can change on a daily basis.”* |

| **Reviewer #4** | **Revision and reply to reviewer’s comment** |
| --- | --- |
| **R4.C1.**  Avoid using words such as ‘suffer’ – suggest ‘experience’ or ‘report’. | The term “suffer” has been removed in the revised first sentence of the abstract. |
| **R4.C2.**  Why does CRF need to be assessed objectively? Treatment can be evaluated on patient self-report similar to pain. Will objective measures change things for the person with CRF – no. | Sensors can produce continuous stream of data to uncover subtle, but potentially important information when patients are still not aware of them to report to the physician. This continuous measurement is impossible with traditional self-assessment systems. Early detection of treatment-related side effects ensures that physicians can intervene before symptoms become severe. Patients may not discuss fatigue with their physicians for a variety of reasons, such as: 1) fear of not receiving maximum cancer treatment due to symptoms, 2) belief that fatigue is an expected and untreatable symptom, 3) concern about being viewed as a “complainer.” Thus, an objective measurement that does not need patients’ report could be beneficial.  The new trend in designing remote patient monitoring and birth of digital biomarkers encourage new solutions to objectively determine measurable digital biomarkers which could be surrogate of physiological and psychosocial metrics including pain, fatigue, stress, depression, etc. We revised the introduction section to better illustrate the concept of ‘digital biomarkers’ (**page 5, line 97**). |
| **R4.C3****.**  Inclusion criteria – why did the participants need to be 55 years or older? | This is a secondary analysis thus inclusion and exclusion criteria were defined based on primary outcomes of the parent study. Please see response to Reviewer 3, Comment **R3.C4.** |
| **R4.C4.**  Why this limited population – only those with CIPN? Not noted in the limitations. | Thank you for this comment. We have revised the limitations (**see page 15, lines 308-310**).  *“Because this study is a secondary analysis, the study population was limited to cancer survivors with CIPN and the study was not powered to clinically validate association between CRF and mobility performance metrics”.* |
| **R4.C5.**  Other differences – number of females – 42% in the non-CRF compared with 25% in the CRF. Frailty differences between groups. Fear of falling – concern about falling has a major impact on mobility. This is not very person-centered in its approach to CRF. | Based on comments from Reviewer #1, we removed the text related to variables which did not achieve a statistically significant level. We have also adjusted results based on fear of falling (in addition to age and BMI) as fear of falling is known to affect mobility performance. We recognize that our study is underpowered to address the effect of all potential confounders and lack of power was acknowledged in the limitation section. |
| **R4.C6.**  The reason why people with CRF have fewer sit to stand transitions is about energy conservation. It takes more energy to move from sit to standing. People with CRF change the activities they do in response to CRF. Studies have shown that cancer survivors do more passive leisure pursuits | We greatly appreciate this insight. With the permission of the reviewer, we added the reviewers’ comment with supporting references as a potential explanation for fewer sit to stand in the CRF group (**Page 14, lines 300-302**). |
| **R4.C7.**  Recommendations for future research missing. Power calculations for an adequately sample size? | The limitations were revised to address issues related to power. Potential future direction has been added the last paragraph of the manuscript **(page 15, line 327).**  ***“****Given the prevalence of CRF among cancer survivors and negative impact on quality of life, larger cohort studies are needed to validate digital biomarkers of CRF, as well as to integrate digital measures of performance mobility into clinical practice and CRF interventions. In addition, future study is warrant to evaluate the sensitivity to change of these digital biomarkers and their ability to track dynamic changes in CRF over time****”*** |
| **R4.C8.**  Correct referencing is needed eg. WHO ICF in the reference list. | Thank you for this note. We have corrected the reference. |
